# Co-occurrence of outlet impingement syndrome of the shoulder and restricted range of motion in the thoracic spine - a prospective study with ultrasound-based motion analysis

**DOI:** 10.1186/1471-2474-11-135

**Published:** 2010-06-29

**Authors:** Christina Theisen, Ad van Wagensveld, Nina Timmesfeld, Turgay Efe, Thomas J Heyse, Susanne Fuchs-Winkelmann, Markus D Schofer

**Affiliations:** 1 Department of Orthopaedics and Rheumatology, University Hospital Marburg, Baldingerstrasse, 35043 Marburg, Germany; 2 Institute of Medical Biometry and Epidemiology, Philipps-University Marburg, Bunsenstrasse 3, 35037 Marburg, Germany

## Abstract

**Background:**

Shoulder complaints, and especially the outlet-impingement syndrome, are a common condition. Among other things, poor posture has been discussed as a cause. A correlation between impingement syndrome and restricted mobility of the thoracic spine (T) has been described earlier, but there has been no motion analysis of the thoracic spine to show these correlations. In the present prospective study, we intended to find out whether there is a significant difference in the thoracic sagittal range of motion (ROM) between patients with a shoulder outlet impingement syndrome and a group of patients who had no shoulder pathology. Secondly, we wanted to clarify whether Ott's sign correlates with ultrasound topometric measurements.

**Methods:**

Two sex- and age-matched groups (2 × n = 39) underwent a clinical and an ultrasound topometric examination. The postures examined were sitting up straight, sitting in maximal flexion and sitting in maximal extension. The disabilities of the arm, shoulder and hand (DASH) score (obtained by means of a self-assessment questionnaire) and the Constant score were calculated. Lengthening and shortening of the dorsal projections of the spine in functional positions was measured by tape with Ott's sign.

**Results:**

On examination of the thoracic kyphosis in the erect seated posture there were no significant differences between the two groups (p = 0.66). With ultrasound topometric measurement it was possible to show a significantly restricted segmental mobility of the thoracic spine in the study group compared with the control group (p = 0.01). An in-depth look at the mobility of the subsegments T1-4, T5-8 and T9-12 revealed that differences between the groups in the mobility in the lower two sections of the thoracic spine were significant (T5-8: p = 0.03; T9-12: p = 0.02). The study group had an average Constant score of 35.1 points and the control group, 85.5 (p < 0.001). On the DASH score the patient group reached 34.2 points and the control group, 1.4 (p < 0.001). The results of Ott's sign differed significantly between the two collectives (p = 0.0018), but showed a weak correlation with the ultrasound topometric measurements (study group flexion/extension: r = 0.36/0.43, control group flexion/extension: r = 0.29/0.26).

**Conclusion:**

The mobility of the thoracic spine should receive more attention in the diagnosis and therapy of patients with shoulder outlet impingement syndrome.

## Background

Impingement syndrome of the shoulder is a term used to describe a number of functional and structural restrictions. It was first described by the American surgeon Charles Neer [1,2] to mean an anatomic narrowing between the head of the humerus and the acromion. In his articles he describes symptoms arising in the shoulder when the upper extremity is internal rotated and lifted, which can be explained by compression of various subacromial structures. The condition progresses in phases and is divided into three stages [1,2]. The mechanical tissue compression that Neer describes below the coracoacromial arch is well known [1,3]. Other factors discussed are a reduced microcirculation in the affected sections of the tendons of the rotator cuff [4-6], with resultant lowering of the metabolism [7]. Zaslav makes a further distinction between outlet impingement and non-outlet impingement, and also regards secondary extrinsic impingement syndrome, such as instability impingement, as a separate condition [8]. Outlet impingement results of changes to the coracoacromial arch, which lead to subacromial compression. A distinction is made between primary changes with anomalies in the form of the acromion, as reported by Bigliani [9], an acromion with its lateral or ventral surface declining steeply, and an acromial bone with acquired alterations, which can include bony excrescences on the acromion and inside the acromioclavicular joint or hypertrophy of the coracoacromial ligament [3].

In day-to-day clinical practice shoulder problems, and especially impingement syndrome of the shoulder, are frequently encountered. The aetiology of the various impingement syndromes has not yet been adequately explained [10]. Suspected predisposing causes are heavy physical work [11-13], long periods of working with the arms above the head [13], or sports requiring the arms to be raised above the head with resultant functional instability [14].

The scapula is linked to the thorax and the spinal column by muscles and also functionally. Insufficient posture and muscular dysbalances are seen as predisposing factors for shoulder dysfunctions [15,16]. A restricted ROM of the thoracic spine could cause or exacerbate an outlet impingement [17-19]. There are several studies that have investigated the restricted range of scapular and shoulder motion e.g. by using three-dimensional scapular kinematics and other methods for motion analysis [20-24]. Other studies described pain decrease in patients with impingement syndromes after thoracic spine (T) manipulation by using manual therapy [25] But there are fewer studies which describe correlations between impingement syndromes and restricted motion of the thoracic spine by showing evidence in the restricted motion of the thoracic spine [18]. Meurer's study is limited in that a plurimeter was used for measurement of the ROM. This method is more reproducible than a normal goniometer, but shows inaccurate values [26].

The aim of this study was to compare the ROM of the thoracic spine in the sagittal plane in patients with outlet impingement syndrome and in patients with no shoulder pathology.

## Methods

### Study group and control group

Following approval from the appropriate ethical committee (file no. 79/08), the study patients and control subjects were recruited from 31 August 2008 to 18 September 2008 in the outpatient clinic of the Department of Orthopaedics and Rheumatology of the University Hospital Marburg, Germany. All patients gave their informed consent to participate in the study. In total 78 patients were investigated, 39 of whom had confirmed outlet impingement syndrome while the other 39 had no shoulder pathology and were used as the control group. The two groups were matched for age and sex. The diagnosis of outlet impingement syndrome was made in each case by the last-named author MDS, who was not involved in the subsequent data collection. The diagnosis was made on the basis of the history elicited, a clinical examination by means of function tests (Neer test, Hawkins-Kennedy test, Speed test, and supraspinatus muscle test), diagnostic X-ray imaging of the shoulder in three planes (a.p., axial, transthoracic), ultrasound and magnetic resonance imaging. Only patients with confirmed outlet impingement caused by osteophytes altering the form of the coracoacromial arch according to Bigliani type II and III acromial shape [9] were included in the impingement group. All patients with concomitant pathologic conditions of the shoulder, such as arthrosis of the joint, a suspected lesion or partial lesion of the rotator cuff, instability, or arthrosis of the acromioclavicular joint, were excluded. Patient selection was subject to stringent inclusion and exclusion criteria (Table 1). The patients in the control group had no shoulder problems; or at least, no shoulder pathology, systemic rheumatic disease, or polyarthrosis was known in this cohort. The same exclusion criteria applied in the control group as in the group of patients with outlet impingement. To confirm that subjects in the control group had no shoulder pathology a detailed history was elicited from each, in addition to which a clinical examination and an ultrasound examination of the shoulders were performed. No further imaging (X-ray, magnetic resonance imaging) was carried out in the control group.

**Table 1:** Inclusion and exclusion criteria following standardised examination of the patient group with outlet impingement syndrome

Inclusion criteria	Exclusion criteria
Outlet impingement syndrome according to Bigliani Type II and III acromion [9]	Systemic rheumatic disease, spinal or thoracic pathology

Shoulder pain for at least 3 months	Operative interventions on shoulder, spine, and thorax

Shoulder pain rated at least 3 on the 10-point visual analogue scale [43]	Shoulder arthrosis, concomitant shoulder pathology (rotator cuff, acromioclavicular joint, or others)

Age 18-80 years	Cognitive impairments

Available documentation of patient consent	Pregnancy

Patients were selected for the outlet impingement group and for the control group by the last-named author (MDS), and patients were examined and the data ascertained independently by the second author (AvW). There was no further blinding of the investigator who was responsible for ascertainment of the data evaluated to assess shoulder function and the subsequent measurements of the range of motion (ROM) in the thoracic spine.

### Assessment of shoulder function

Shoulder function was ascertained in all patients with the Constant score [27,28] and the DASH (disabilities of the arm, shoulder and hand) questionnaire [29]. The Constant score is a test procedure with mixed weightings and is used as an instrument for the assessment of shoulder function. The DASH score is calculated from a self-report questionnaire and ascertains the test persons' subjective perception of their current condition. Greater dysfunctions of the shoulder is imaged by a high DASH score and lower Constant score, whereas a good shoulder function is indicated by a lower DASH score and a higher Constant score. In addition to these data, demographic data and general characteristics, such as sex and handedness, were also recorded. The basic descriptive characteristics of the subjects are given in table 2.

**Table 2:** Subject characteristics (M = male; F = female; L = left-side dominance; R = right-side dominance)

	Study group	Control group
	(n = 39)	(n = 39)
**Mean Age** **(Range/median)**	56.6 (38 - 77/55)	56.1 (38 - 79/54)

**Sex**	23M/16 F	23 M/16 F

**Dominant side**	2 L/37 R	3 L/36 R

**Dominant side affected**	25	not relevant

### Assessment of the thoracic spine function

**Figure 1:**
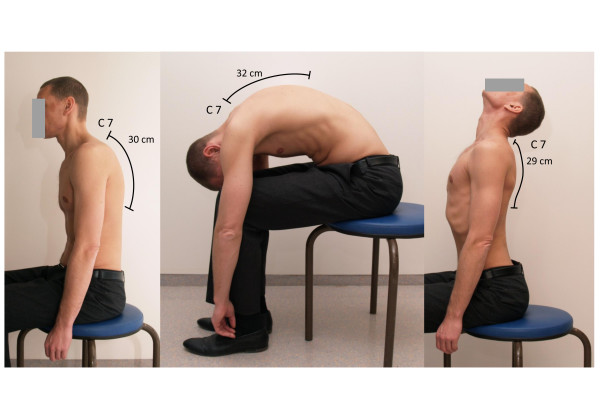
**Ott's sign**.

Ott's sign [30] was carried out to measure the ROM of the thoracic spine in the sagittal plane. This test is used to assess to what degree the thoracic spine can unfold. For measurement, the most prominent spinous process (cervical spine (C7)) is detected and marked in the relaxed sitting subject. The landmark 30 cm caudal is marked as well. Then the changes in the length when the patient bends maximal forward and maximal backward are measured with a tape. Lengthening of 2-4 cm and shortening of 1 cm are normal values (Fig. 1).

Ultrasound topometry is a measurement for assessment of motion analysis system based on surface mounted transmitters in a three-dimensional space and it was carried out with the CMS 20 measurement system (Zebris Medical GmbH, Isny, Germany). The system includes appropriate measuring instruments (ultrasound wand PS33-1 with two ultrasound transmitters, reference marker PR-1, triple ultrasound microphone MA-XX on a floor-standing tripod) [31,32]. Measuring and data ascertainment were achieved with the program WinSpine 2.2.3 (Zebris Medical GmbH, Isny, Germany).

The measuring method is based on determination of the spatial coordinates of the ultrasound transmitters by a fixed system of three microphones whose positions are relative to a fixed system standing close by. The ultrasound transmitters send continuous pulses. Using triangulation, the measurement was derived from the time delay between the ultrasound pulses measured at a sample rate of 20 Hz, which is a standard frequency for static positions [33-35]. The ultrasound pulses are then calculated and imaged through the system. The analyzed data can then be displayed graphically. Former studies, measuring the active Rom of the cervical and lumbar spine showed reproducible results using the Zebris CMS measurement system [33,34,36]. The Zebris spine motion analysis shows reliable and comparable measurements of cervical spine ROM compared to other systems [37]. There has been less investigations of the ROM of the thoracic spine because CMS 20 is only approved for investigation of hand-arm-motions, cervical spine and mandibular joint motion analysis. To determine the reliability of the Zebris testing of the thoracic spine, a healthy sample of 19 volunteers were investigated and took part in a test-retest-reliability design. The test-retest-reliability was assessed by repeating the measurement three times of each subject. To establish the details of the various segments of the spine, it is measured with the reference marker from C7 down to the sacrum several times. The average data is used for subdivision of the segments. The relative size of these segments is given through the data file pointer.ini (c:\programme\zebris\WinSpine). The calculation of the physical size of the various segments of the spine is carried out using the following form: length of the spine in mm × relative size/sum of all relative vertebral bodys.

**Figure 2:**
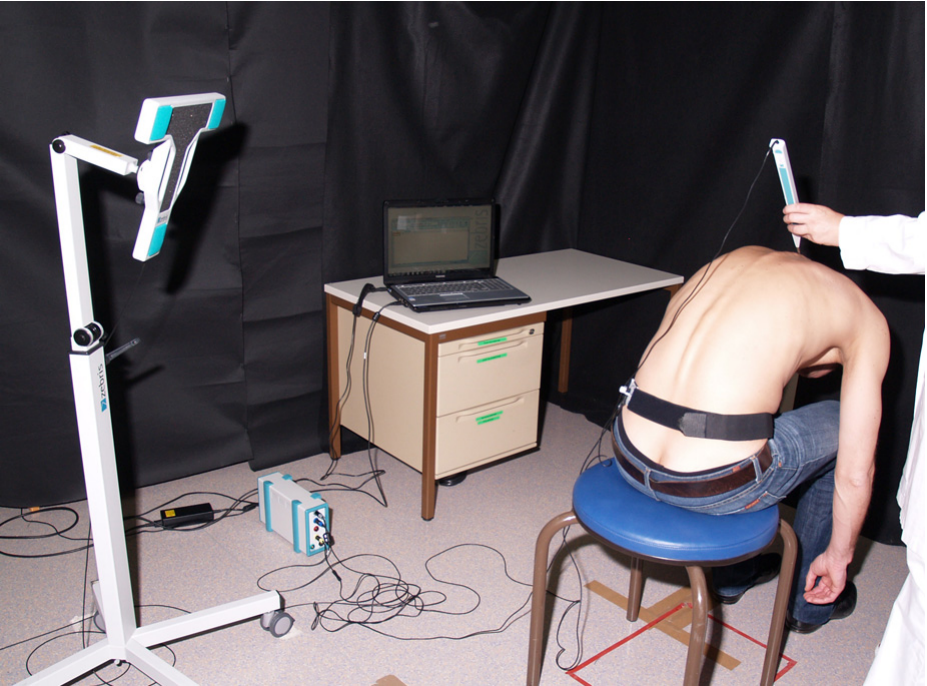
**Experimental set-up of the ultrasound-based motion analysis**.

For ultrasonic measurement, anatomic landmarks were marked as reference points at the relaxed standing patient: interspinal space from C7-T1, beginning from the most prominent spinous process, the dorsolateral angle of the acromial bone bilaterally and the spina iliaca posterior superior bilaterally. These points were scanned with the reference marker PR-1. The scanning process was carried out by moving the reference marker along the spine, so that the distance between the landmarks was measured. The ultrasound microphone markers receive signals from the transmitters located in the measuring unit. All measuring devices are positioned on exactly defined places and the positions were not changed during the investigation (Fig. 2). For the motion of the thoracic spine to be measured, the patient sat stripped to the waist with the upper body erect. No corrections were made to the patients' subjective perception of an erect sitting position (static kyphosis). Measurements were taken in standardised conditions directly on the skin over the spinous processes. Each measurement was repeated three times. There was no validation of the Zebris testing through functional X-ray during motion. The study group of Strimpakos showed that there are only small differences in the ROM analysis of the neck between the Zebris motion system compared to X-ray [38]. That confirms the validity of the method used.

Before Ott's sign was carried out and before the ultrasound topometric measurement a 'warm-up' programme was implemented with the patient already seated. The purpose of the warm-up was to correct articular motion restrictions in the spinal column that were attributable to inadequate synovial lubrication before the measurements were made, so as to keep any distortion of the results to a minimum.

To determine the maximal flexion possible the seated patient tucked in his/her head and leant down towards the floor with the arms hanging downward against the sides of the body. For maximal extension the patient was requested to make a sway back while doing his/her best to look up at the ceiling. In addition to the determation of the overall ROM in the thoracic spine, the thoracic spine was divided into three segments (T1-4; T5-8; T9-12) and the ROM in each was also measured. This was done by applying predetermined reference points and calculating the shape of the spine using the norms provided by Messrs. Zebris for the individual sizes of the vertebrae.

The data recorded were imported into MS Excel 2007 as raw data in ASCII format. The following data were prepared from the test values:

• the resting position (static kyphosis), defined as the erect sitting posture in which each individual patient felt comfortable

• the flexion values, corresponding to the maximal ROM in forward inclination

• the extension values, corresponding to the maximal ROM in backward inclination

### Study design

The main task in this study was to examine whether there were significant differences in the thoracic sagittal ROM between patients with outlet impingement syndrome of the shoulder joint and patients with no shoulder pathology. The null hypothesis (H_0_) and the alternative hypothesis (H_1_) were set up:

H_0_: The ROM of the thoracic spine in the sagittal plane is not altered in patients with outlet impingement of the shoulder.

H_1_: The ROM of the thoracic spine in the sagittal plane is altered in patients with outlet impingement syndrome of the shoulder.

A secondary task was to find whether there was a correlation between the result of Ott's sign and the ultrasound topometric measurements.

Case number calculation was carried out before commencement of the study with the aid of the unpaired t-test for an expected difference of 10° in the ROM of the thoracic spine, with a standard deviation of 15.1° estimated from results in the literature [17,18], yielded a number of 37 needed per group to achieve 80% power with a two-sided significance level of 5%.

### Statistics

For the descriptive analysis, the means and standard deviations and the minimal and maximal values were given. The differences between the two groups were examined with the aid of Welch's two-sided t-test, and/or the two-sample t-test in the case of approximately equal variances. Before performance of the t-test the assumption of normal distribution was tested with the Kolmogorov-Smirnov test. In all comparative investigations the 95% confidence interval (CI) is given in addition. The level of significance selected was 5%. All correlations were calculated according to the Pearson method.

## Results

### Test retest reliability

The test retest reliability was made up of 19 volunteers without disorders of the spine. The average age was 33.6 (24-67) years. Each subject was investigated two times at intervals of one hour. Each measurement was repeated three times. The average value was detected and the Pearson correlation coefficient was calculated. The results are presented in Table 3.

**Table 3:** Pearson correlation coefficient for different positions of the thoracic spine

Static position	0.90
**Flexion**	0.74

**Extension**	0.78

**ROM**	0.87

**Figure 3:**
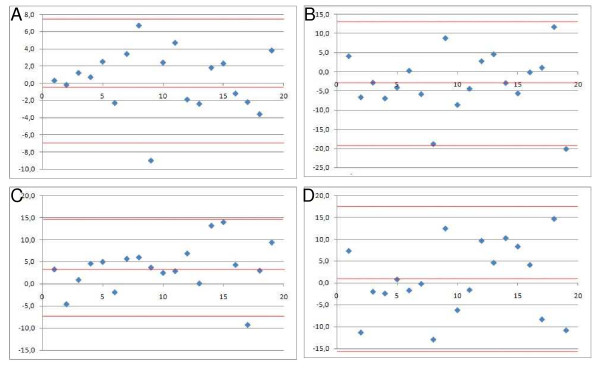
**A: BA-plot static position, B: BA-plot flexion, C: BA-plot extension, D: BA-plot ROM**.

The visualisation of the correlation coefficient by using a BA-plot is presented in figure 3[39,40]. The calculated values are shown in Table 4.

**Table 4:** Detailed results of the BA-plots

	Static position	Flexion	Extension	ROM
**Mean value of the differences**	0.4°	2.9°	3.7°	0.8°

**Mean value +2 SD**	7.6°	13.1°	14.7°	17.4°

**Mean values -2 SD**	-6-8°	-18.9°	-7.3°	-15.8°

### Study group

The study group was made up of 39 study participants with an average age of 56.6 ± 10.2 (38-77) years. The sex distribution was 23 female and 16 male patients. Two patients were left handed. In 25 cases the shoulder affected was on the dominant side.

### Control group

The control group was made up of 39 patients with an average age of 56.1 ± 10.3 (38-79) years. Twenty-three women and 16 men were allocated to the control group. Three subjects were left handed.

### Shoulder function

In the study group the mean DASH score was 34.2 ± 19.8 (1-79) points; for the control group the mean was 1.4 ± 2.0 (0-10) points.

The mean Constant score in the study group was 35 ± 16.4 (12 77) points. In the control group the mean score was 85.5 ± 6.0 (73-98) points.

Both the DASH score and the Constant score differed significantly between the two groups (p < 0.001 for each).

### Ott's sign

In the study group the mean of the summated values was 3.7 ± 1.2 (1.9-8.9) cm. In the control group the mean of the summated values was 4.6 ± 1.2 (2.0-7.9) cm. The difference between the two groups is significant (p = 0.0018; CI: 0.5-1.3).

### Ultrasound topometric measurements

The results for measurement of ROM in the thoracic spine are presented in Table 5.

**Table 5:** Detailed results in the study group and in the control group (SD=standard deviation; CI = confidence interval; SG = study group; CG = control group; ROM = range of motion)

		Mean	Minimum	Maximum	SD	CI	p-value
**Static kyphosis**	**SG**	**45.9°**	**5.7°**	**66.3°**	**10.8°**	**42.5-49.3**	**0.66**
	
	**CG**	**44.8°**	**27.2°**	**69.2°**	**10.6°**	**41.5-48.1**	

**Flexion**	**SG**	**15.2°**	**4.6°**	**33.6°**	**9.4°**	**12.3-18.1**	
	
	**CG**	**19.8°**	**2.6°**	**37.8°**	**9.5°**	**16.8-22.8**	

**Extension**	**SG**	**12.7°**	**5.9°**	**29.3°**	**8.0°**	**10.2-15.2**	
	
	**CG**	**14.9°**	**0.6°**	**32.9°**	**7.6°**	**12.5-17.3**	

**Functional ROM**	**SG**	**28.0°**	**3.1°**	**50.5°**	**12.7°**	**24-32**	**0.01**
	
	**CG**	**34.6°**	**12.9°**	**55.5°**	**9.6°**	**31.6-37.6**	

Investigation to check for differences between the two groups revealed that they did not differ significantly in static kyphosis (p = 0.66; CI:-3.7-5.9). In total we measured a difference in the static kyphosis of 1° (45.9° in the study group and 44.9° in the control group). In the evaluation of the functional total ROM of the thoracic spine a significant difference of 6.6° was demonstrated (p = 0.01; CI:1.5-11.7).

The functional mobility in the T1-4 segment of the thoracic spine did not differ significantly between the groups (p = 0.20; CI:-1.3-4.7).). The actual measurement in degree of motion was 8.0° ± 6.2° in the control group and 6.3° ± 7.2° in the patient group. In total, it is a difference of 1.7° in the ROM of the segment T1-4.

In the other segments, T5-8 and T9-12, significant differences were documented between the groups (p = 0.0308; CI: 0.03-4.2 and p = 0.02; CI:0.5-4.9, respectively). In segment T5-8, we measured 9.9° ± 3.9° in the control group and 7.8° ± 5.1° in the patient group. In total it is a difference in ROM of 2.1°. In segment T9-12 we detected the most relevant findings. The ROM of the segments T9-12 in the control group was 16.7° ± 5.3° and 14° ± 4.4° in the patient group. The total difference of the ROM in this segment was 2.7°.

The correlations between the results of the Ott sign and of ultrasound topometry showed only a weak connection with values between 0.26 and 0.43, regardless of whether the tests were performed in flexion or in extension. This comparison, then, revealed no significant correlation (Table 6).

**Table 6:** Correlation between the results of the Ott sign and the ultrasound topometric measurements (r = correlation coefficient; SG = study group; CG = control group; ROM = range of motion)

r	SG	CG
**Flexion**	0.36	0.29

**Extension**	0.43	0.26

**Functional ROM**	0.18	0.36

When the Constant score and the functional overall ROM were examined for correlation, a negative correlation (r = -0.29) was found in the study group. Comparison of the DASH score and the functional total ROM revealed a non significant correlation (r = 0.12).

## Discussion

The ultrasound topometric examination of the thoracic spine made it possible to detect a significantly restricted ROM in the sagittal plane in the outlet impingement group. It was also shown that the posture of the thoracic spine in outlet impingement patients was not significantly different from that in the group with no shoulder pathology. Significant differences between the two groups were found for both functional ROM. On comparison of results recorded in the two groups in individual segments of the thoracic spine significant differences were also observed. This applies the functional analysis for segments T5-8 and T9-12, which means a positive answer to the main question asked in the study: a difference was demonstrated in the thoracic spine mobility of patients with outlet impingement as against patients with no shoulder pathology. This may be used as background knowledge for the treatment of patients, because ultrasound motion analysis is not a routinely used clinical diagnostic procedure and the difference we found that show statistical significance, might be easy to detect in the routinely performed clinical examination. The ultrasound topometric measurement system is in contrast to the easy, realisable and practicable ott test, much more difficult in the setup of the system and of course it it much more expensive and less practicable. Both measurement systems are easily associated with errors according the setup and accomplishment.

The secondary question was whether there was a correlation between the results of the Ott sign and of the ultrasound measurements. In answer to this it was shown that there was a significant difference between the groups in the results as far as the Ott sign is concerned. Regardless of the direction of motion, the correlation coefficients showed that with values between 0.26 and 0.43 there was only a weak correspondence between the results of the Ott sign and of the ultrasound topometric measurements. Although Ott's sign highlights the differences between the outlet impingement group and the control group, it is too inaccurate compared with ultrasound topometry. This means that Ott's sign can only be used as an indicator of restrictions in the mobility of the thoracic spine. Ott's sign does not allow reliable statements about the amplitude of motion of the thoracic spine in flexion and extension or about the total ROM.

The supposition that it is mainly the mobility of the thoracic spine, and not just the posture in this section of the spine, that has some role in outlet impingement syndrome of the shoulder has been confirmed by this study. However, it is not possible on the grounds of this study to clarify whether the altered spinal mobility in the thoracic section is caused by the outlet impingement syndrome or whether the impingement arises as a result of the altered mobility. The idea of restricted mobility of the thoracic spine as an aetiological factor in the development of outlet impingement syndrome derives credence from the absence of any significant correlation of the DASH score and the Constant score and the total functional ROM. If outlet impingement syndrome of the shoulder were responsible for the restrictions in the mobility of the thoracic spine it might be expected that with increasing severity of the shoulder condition the consequences for the thoracic spine would become increasingly clear. No such connection is obvious.

The significant group differences in the ultrasound topometric measurements are not very great in absolute quantitative terms, but are quite significant when seen in context. For functional mobility the means in the study group and in the control group differed by 6.6°. This means that in the study group the thoracic spine was less mobile by 19.9% than in the control group. This highlights the necessity for accurate measuring systems, as subjectively these differences cannot be perceived sufficiently clearly.

In a prospective study in 100 subjects, Meurer were able to show that the thoracic mobility in all planes was significantly less in the shoulder patients than in the control group [17,18]. The mobility of the thoracic spine was measured with a Rippstein plurimeter. In the case of mobility in the sagittal plane, the mobility of those with no shoulder pathology was significantly greater (p < 0.0082) than that of all the shoulder patients. One criticism that must be levelled at this study is that the diagnosis of impingement syndrome was not clearly defined [17,18].

Meurer's ultrasound topometric measurements have been largely confirmed by the present study [17,18]. A point of difference between Meurer's study and the present one is that the evaluation of our ultrasound topometric measurements allowed separation of the results for different sections of the spine. The biggest differences between the groups were found in the middle (T5-8) and lower (T9-12) segments of the thoracic spine.

The lacking control of the reference points marked for the ultrasound measurements and the Ott sign could be one limitation. The software used was set ex works in such a way that the superior posterior iliac spines were used as reference points for S3. A study by McGaugh has shown that this is not necessarily correct [41].

As this study cannot determine whether the restricted mobility of the thoracic spine is implicated in the aetiology of outlet impingement syndrome or whether it is a consequence of the impingement syndrome, further studies are needed to cast more light on this question. One possibility would be to have a study designed as a therapy study, with test groups 'standard treatment, shoulder' and 'standard treatment, shoulder + manual treatment of the thoracic spine'. If there are obvious differences between the groups we can assume that the thoracic spine is one factor that is aetiologically responsible for the development of impingement syndrome. This question was taken up by Bergman, but the additional treatment of the spine was only administered to the cervical segment and the upper thoracic segment of the spine [42]. The symptoms did improve in the group with additional manual therapy. It should be borne in mind, however, that the degree of improvement in the symptoms was dependent on the manual therapist and a variance of 14-67%, depending on therapist, in the result of treatment was reported [42].

## Conclusion

The use of ultrasound topometry made it possible to show altered sagittal mobility of the thoracic spine in patients with an outlet impingement syndrome of the shoulder compared with patients who had no shoulder pathology. The impaired mobility was localised in the middle and lower segments of the thoracic spine. The two groups did not differ in static kyphosis. The implication of this for clinical practice is that shoulder impingement patients should be examined for impaired spinal mobility at least in the thoracic segment. Concomitant treatment for the impaired mobility of the thoracic spine is advisable.

## Abbrevations

ROM: range of motion; DASH: disabilities of the arm, shoulder and hand; T: thoracic spine; C: cervical spine; H: Hypothesis; CI: confidence interval.

## Competing interests

The authors declare that they have no competing interests.

## Authors' contributions

CT drafted the manuscript and was responsible for formulation of the manuscript as well as for data interpretation. AvW and MDS were responsible for the study design. AvW was also responsible for data collection, the questionnaire survey and data interpretation. MDS was involved in drafting the manuscript. NT did the statistical analysis of the study and was responsible for the statistical methods used in the study. TE and TJH took part in the formulation of the study and were also involved in drafting the manuscript. SFW participated in the description of background knowledge. All authors critically read, revised and finally approved the manuscript.

## Pre-publication history

The pre-publication history for this paper can be accessed here:

http://www.biomedcentral.com/1471-2474/11/135/prepub
